# Oculomotor abnormalities indicate early executive dysfunction in prodromal X-linked dystonia-parkinsonism (XDP)

**DOI:** 10.1007/s00415-023-11761-8

**Published:** 2023-05-16

**Authors:** Renana Mertin, Cid Diesta, Norbert Brüggemann, Raymond L. Rosales, Henrike Hanssen, Ana Westenberger, Julia Steinhardt, Marcus Heldmann, Hans T. S. Manalo, Jean Q. Oropilla, Christine Klein, Christoph Helmchen, Andreas Sprenger

**Affiliations:** 1 Department of Neurology, University Hospital Schleswig-Holstein, University of Lübeck, Ratzeburger Allee 160, 23538 Lübeck, Germany; 2 Center of Brain, Behavior and Metabolism (CBBM), University of Lübeck, Lübeck, Germany; 3 Makati Medical Center, Makati City, Philippines; 4 Asian Hospital and Medical Center, Manila, Philippines; 5 Institute of Neurogenetics, University of Lübeck, Lübeck, Germany; 6 Department of Neurology and Psychiatry, University of Santo Thomas, Manila, Philippines; 7 Institute of Psychology II, University Lübeck, Lübeck, Germany

**Keywords:** X-Linked dystonia-parkinsonism (XDP), Smooth pursuit, Anti-saccades, Memory-guided saccades, Prodromal

## Abstract

**Background:**

X-Linked dystonia-parkinsonism (XDP) is a movement disorder characterized by the presence of both dystonia and parkinsonism with one or the other more prominent in the initial stages and later on manifesting with more parkinsonian features towards the latter part of the disease. XDP patients show oculomotor abnormalities indicating prefrontal and striatal impairment. This study investigated oculomotor behavior in non-manifesting mutation carriers (NMC). We hypothesized that oculomotor disorders occur before the appearance of dystonic or parkinsonian signs. This could help to functionally identify brain regions already affected in the prodromal stage of the disease.

**Methods:**

Twenty XDP patients, 13 NMC, and 28 healthy controls (HC) performed different oculomotor tasks typically affected in patients with parkinsonian signs.

**Results:**

The error rate for two types of volitional saccades, i.e., anti-saccades and memory-guided saccades, was increased not only in XDP patients but also in NMC compared to HC. However, the increase in error rates of both saccade types were highly correlated in XDP patients only. Hypometria of reflexive saccades was only found in XDP patients. Initial acceleration and maintenance velocity of smooth pursuit eye movements were only impaired in XDP patients.

**Conclusions:**

Despite being asymptomatic, NMC already showed some oculomotor deficits reflecting fronto-striatal impairments, typically found in XDP patients. However, NMC did not show saccade hypometria and impaired smooth pursuit as seen in advanced Parkinson’s disease and XDP, suggesting oculomotor state rather than trait signs in these mutation carriers. Neurodegeneration may commence in the striatum and prefrontal cortex, specifically the dorsolateral prefrontal cortex.

**Supplementary Information:**

The online version contains supplementary material available at 10.1007/s00415-023-11761-8.

## Introduction

Eye movement abnormalities crucially help to distinguish and identify different parkinsonian syndromes [1, 33], e.g., Parkinson’s disease (PD) from multiple system atrophy with predominant parkinsonism (MSA-P) and progressive supranuclear palsy (PSP). PD-typical eye movements abnormalities, however, can already be found in non-manifesting carriers of PD-causing mutations, e.g., in *Parkin* [36] or *PINK1*, in the absence of a motor phenotype [25]. Not only imaging parameters [47] but also subtle behavioral parameters may help to identify brain regions already involved in the very initial neurodegeneration in the prodromal stage of the disease. X-Linked dystonia-parkinsonism (XDP) is an adult-onset neurodegenerative movement disorder characterized by the transition of initially focal and rapidly generalizing dystonia into parkinsonism after 10–15 years in most patients [28, 48]. XDP is endemic to the island of Panay, Philippines, due to a genetic founder mutation [29]. Men are mainly affected due to the X-linked inheritance with an assumed complete penetrance. The genetic cause is a SINE-VNTR-Alu (SVA) retrotransposon insertion in the TATA-box-binding-protein-associated-factor-1 gene (TAF1). This insertion contains a hexanucleotide repeat ((CCCTCT)n), the number of which correlates with the age at disease onset and disease severity [6, 56]. The resulting reduced expression of *TAF1* is associated with neurodegeneration mainly in the striatum and to a lesser degree in the pallidum [10, 18] with increased iron accumulation [11, 20] and widespread microstructural changes of the white matter [6, 10]. Striatal atrophy correlates with disease duration and the neurodegenerative process follows an antero-posterior gradient with a stronger involvement of the rostral striatum [18, 21]. Striatal degeneration was also found to be associated with decreased post-synaptic D2-receptor density [11]. In addition, extrastriatal gray matter changes have also been identified [3], including the cerebellum [20] and frontal and temporal cortex [6, 20] corresponding to astro- and microgliosis of the prefrontal cortex (PFC) in post-mortem investigations [40]. Structural connectivity of the striatal striosome compartment (next to the striatal matrix the major component containing projections neurons) was not decreased but increased with the anterior insula [6]. Pallidal neurostimulation markedly improves generalized dystonia in particular if caudate atrophy is modest [9].

Striatal atrophy and iron accumulation in XDP resembles Huntington’s disease (HD) [3, 13]. TAF1 variants seem to be a likely determinant of selective striatal vulnerability in both diseases [24]. Similar to HD [13], striatal atrophy was recently demonstrated in non-manifesting mutation carriers (NMC) by voxel-based morphometry and subcortical volumetry arguing for a prodromal phase with significant neurodegeneration prior to the onset of the overt movement disorder [19].

Clinically, the motor phenotype of XDP is different from classical HD; its late parkinsonian phenotype can be mistaken for MSA-P, the Westphal variant of HD or PD. Likewise, oculomotor abnormalities in the parkinsonian stage of XDP patients with long-lasting disease resembled those of patients with MSA-P and—to a lesser degree—PD [51]. Several features, however, also showed an overlap with HD [39]: XDP patients showed increased error rates of anti-saccades which correlated with the reduction of (i) the volume of the putamen as well as (ii) the volume and cortical thickness of the dorsolateral prefrontal cortex [51]. Similar to HD, vertical saccade latency was increased. Saccade velocity, however, was not slowed in manifest XDP, in contrast to HD [30] and PSP.

However, it remained unclear whether these oculomotor abnormalities reflect changes of an advanced disease stage or correspond to alterations that occur early in the disease process. Such knowledge could help to elucidate the pathological mechanism of the unique transition of generalized dystonia into a parkinsonian syndrome. In HD, increased error rates in the memory-guided saccade and anti-saccade tasks reflecting the deficient capacity to suppress unwanted saccades were already found in the pre-manifest stage [5].

Therefore, we studied eye movements in NMC of the *SVA* retrotransposon insertion, i.e., when striatal neurodegeneration is likely to already exist and before subjects had clinical signs or symptoms. The penetrance of the pathogenic variant is very likely complete [27] indicating that NMC are highly prone to develop the disease. As generalized dystonia is not associated with eye movement abnormalities, eye movement recording in NMC may be used to identify brain mechanism in the very early parkinsonian manifestation of the disease.

We, therefore, compared oculomotor behavior in the prodromal phase of XDP with healthy controls (HC) and XDP patients **a**nd related behavioral group differences to morphological abnormalities (voxel-based morphometry and subcortical volumetry imaging) and gait/postural stability parameters of these participants [19, 52]. This approach allows us to replicate oculomotor findings by comparing them with a different, previously described XDP cohort using the same technique [51]. Specifically, we tested whether NMC already show saccade abnormalities (anti-saccade, memory-guided saccades) reflecting fronto-striatal dysfunction that we found in XDP patients. As pronounced neurodegeneration (striatal atrophy) have already been found in newly diagnosed XDP patients, prodromal signs in NMC are plausible and have already been shown for gait parameters [52].

## Materials and methods

### Participants

Our study population consisted of 61 male Filipinos between the ages of 23 and 57 including 20 XDP patients, 13 NMC, and 28 HC. For NMC recruitment, 32 male relatives of previously genetically identified XDP patients underwent genetic analysis via genomic DNA testing from peripheral blood samples. If genetic analysis revealed a SVA retrotransposon insertion in the TAF1 gene, the hexanucleotide repeat number was extracted [7]. This was the case for 14 participants, the other 28 healthy males served as the control group. Genetic testing was done after the experiments allowing a double-blind study design. One NMC had a XXY karyotype (Klinefelter’s syndrome) and was excluded from the oculomotor examination. For correlation, we obtained the repeat number and the genotypes at the three single nucleotide polymorphisms tagging the genetic modifiers of age at onset [26] in NMC and XDP patients. Based on the repeat number and the above-mentioned genotypes, we estimated age at disease onset in NMC [19, 52]. In addition to the oculomotor tasks listed below, all participants underwent MRI-imaging examination as well as various clinical assessments (MOCA-P, BFMDRS, MMSE, MDS-UPDRS, HADS-D/A, FAB, gait parameters). Demographic, genetic and clinical features of the groups can be found in Table 1 of the supplementary material. NMC were classified “non-manifesting” in case they did not report any neurological symptoms. In the next step, a neurological and clinical oculomotor examination was performed to exclude neurological signs that would indicate a clinical diagnosis of a movement disorder. Importantly, there was no evidence for clinically relevant cognitive dysfunction or neuropsychiatric symptoms.

**Table 1:** Amplitude-matched latencies for horizontal pro-saccades (group comparisons)

Comparison	5°	10°	15°
XDP vs. HC	*Z = *− 2.607; *p = ***0.008**	*Z = *− 3.350; *p < ***0.001**	*Z = *− 1.081; *p = *0.284
NMC vs. HC	*Z = *− 1.727; *p = *0.084	*Z = *− 2.524; *p = *0.010	*Z = *− 0.595; *p = *0.610
XDP vs. NMC	*Z = *− 0.490; *p = *0.650	*Z = *− 0.901; *p = *0.374	*Z = *− 0.721; *p = *0.494

### Experimental setup and oculomotor tasks

Eye movements were recorded via a portable, video-based eyetracking system with binocular recording at a sampling rate of 500 Hz. The exact experimental setup of stimuli as well as the recording program is documented in the supplementary material and similar to our previous study [51].

The participants were asked to perform reflexive saccades (horizontal, vertical, amplitudes; 5°, 10°, 15°, gap 200 ms), volitional saccades (anti-saccades, memory-guided [MGS]) and smooth pursuit eye movements ([SPEM], sinusoidal and foveopetal step-ramp pursuit [46]). Experimental procedures for all paradigms are described in the supplementary material and are similar to our previous study on XDP patients [51].

To rule out gaze-evoked nystagmus or square wave jerks, each participant was asked to fixate a target stimulus for 10 s at gaze straight ahead position as well as at 15° position in horizontal and vertical direction.

Participants, who did not understand English, were instructed by native Filipinos to rule out any instrumental misunderstanding of the instruction.

### Imaging analysis

T1-weighted and susceptibility-weighted images were surveyed using a 1.5 T Magnetom Aera, Syngo MR D13 (Siemens Healthcare, Erlangen [GER]). Data were analyzed using voxel-based morphometry and subcortical volumetry, for details see [19]. Oculomotor findings were correlated with striatal and pallidal areas, similar to [51].

### Quantitative balance and gait analysis

Balance and gait parameters were quantified using a wearable APDM’s Mobility Lab System™ (Portland, OR). Participants underwent different conditions with increasing difficulty, for details see [52]. Oculomotor findings in NMC were tested for correlations with the parameters that turned out to be most sensitive to delineate HC from NMC subjects: standing on foam with eyes closed; walking at maximum speed for 2 min) [52].

### Data and statistical analysis

Eye movement data were analyzed using custom made software in Matlab (R2019b, The MathWorks Inc., Natick, MA, USA). Saccade and blink detection was checked interactively. Furthermore, each trial of the different tasks was manually verified. Eye movement performance was calculated as gain values for saccade tasks (eye amplitude/target amplitude) and smooth pursuit tasks (eye velocity/target velocity) (0.2 Hz (20°/s)). Saccade latency variability was stated as coefficient of variation (ratio of SE [see below] and mean of latency) for each target amplitude (5°, 10°, 15°). Saccade peak velocity was derived from a main sequence fit procedure using *fminsearch* function within Matlab® and calculated for 10°, 15°, and 20° amplitudes. In the anti-saccade task, reflexive (error-) saccades towards the target were counted and related to the amount of executed trials (anti-saccade error rate, AER). In the memory-guided saccade (MGS) task, three memorization times (MT, MT1 = 2 s, MT2 = 3 s, MT3 = 4 s) were compiled. Erroneous reflexive saccades towards the flashed target (rate of reflexive erroneous saccades, RES) were counted similar to the AER and related to the amount of executed trials (for details, see [51]). Disease duration in XDP patients was calculated as the subtraction of the age at disease-related symptom onset from the current age during examination.

If normality test failed, non-parametric tests (Kruskal–Wallis-, Mann–Whitney *U* test) were used for analyzing the data, otherwise conventional comparisons for groups (e.g., variance analysis (ANOVAs) or two groups or conditions (*t*-test) are reported. For multiple variables with missing normality, data were rank transformed and subsequently analyzed by ANOVA and Student’s *t*-test. In some ANOVAs, sphericity requirement was violated. Therefore, we report *p* values with Greenhouse–Geisser correction but report degrees of freedom (*df*) uncorrected to show the factorial analysis design. Significance levels of post hoc tests were Bonferroni corrected for multiple testing. Correlation coefficients were analyzed using Spearman’s Rho. Statistical differences were regarded as significant for values *p < *0.05. Box plots show the median (midline) and a box (25th to 75th percentile of the distribution). Error bars indicate the minimum/maximum value or 1.5*interquartile range if outliers (circles) are present.

## Results

Clinical score parameters were not correlated with any of the oculomotor behavioral parameters listed below.

There was no main effect of target direction in any task nor an interaction between target direction and group; therefore, data were averaged for direction.

### Pro-saccades

#### Horizontal pro-saccades

XDP patients (155 ± 5 ms) and NMC (154 ± 6 ms) did not differ in latencies, irrespective of the amplitude. Latencies of both groups also did not differ from large amplitudes (15°) of healthy controls while they were shorter for small amplitudes (5°: *Χ*^2^(2) = 7.653, *p = *0.022; 10°: *X*^2^(2) = 13.892, *p = *0.001). Post-hoc tests (Mann–Whitney *U* test) showed shorter saccade latencies for XDP patients for each amplitude, NMC only for the 10° amplitude, compared to HC. Importantly, post hoc tests revealed no differences between XDP patients and NMC. Table 1 shows group comparisons of amplitude-matched saccade latencies (z-values) and their significant differences. The variability of latency differed between groups regarding 5° amplitude (X^2^(2) = 8.005, *p = *0.018) but neither for the 10° (X^2^ = 2.339, *p = *0.310) nor the 15° amplitude (X^2^(2) = 1.579, *p = *0.454). Post-hoc tests for the 5° amplitude revealed an increased variability of latency of XDP patients compared to HC (*p = *0.009) and a trend for NMC vs. HC (*p = *0.056) but not between themselves (*p = *0.769).

**Fig. 1:**
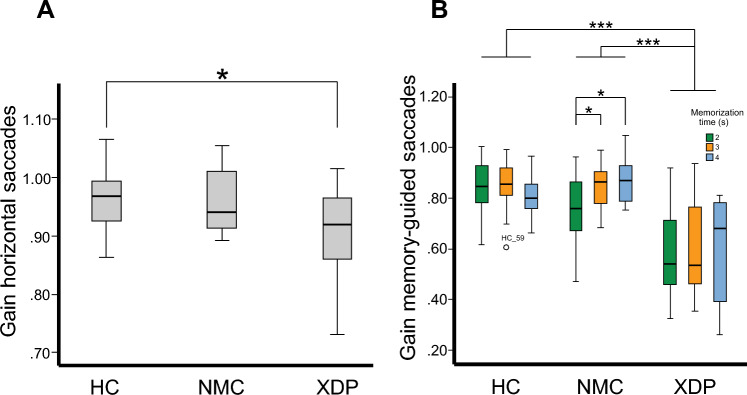
Amplitude gain for horizontal pro-saccades (**A**) of XDP patients, NMC and HC. XDP patients showed saccade hypometria compared to HC. Group comparison revealed no difference between NMC and HC. The horizontal saccade gain (**B**) during the memory-guided saccade task is displayed as a function of the three memorization intervals (2, 3, 4 s) for the three groups of participants (XDP, NMC, HC). The saccade hypometria of XDP patients between groups (compared to NMC and HC) was independent of the memorization intervals. However, saccade gain slightly increased with increasing memorization intervals in NMC and XDP patients (trend) that was not found in healthy control subjects. *p* values: * < 0.05; ** < 0.01; *** < 0.001. XD*P  *X-linked dystonia-parkinsonism, *NMC* non-manifesting mutation carriers, *HC* healthy control subjects

Saccade accuracy (gain) was reduced in XDP patients (0.90 ± 0.8) but not NMC (0.96 ± 0.06) compared to HC (0.96 ± 0.05; *F*(2,51) = 4.26, *p = *0.019; XDP vs. HC: *p = *0.021, XDP vs. NMC: *p = *0.091, NMC vs. HC: *p = *1.0, Fig. 1A), i.e., only XDP patients but not NMC showed saccade hypometria.

#### Vertical pro-saccades

There was no significant difference between groups concerning latency (*p = *0.219) and gain (*p = *0.912). Saccade accuracy (gain) was not different between groups.

#### Main sequence of saccades

Peak horizontal saccade velocity was not different between the groups, irrespective of the amplitude: 10°-saccades (*Χ*^2^(2) = 0.778; *p = *0.674), 15°-saccades (*Χ*^2^(2) = 0.805; *p = *0.669) and 20°-saccades (*Χ*^2^(2) = 0.958; *p = *0.619).

### Voluntary saccades

**Fig. 2:**
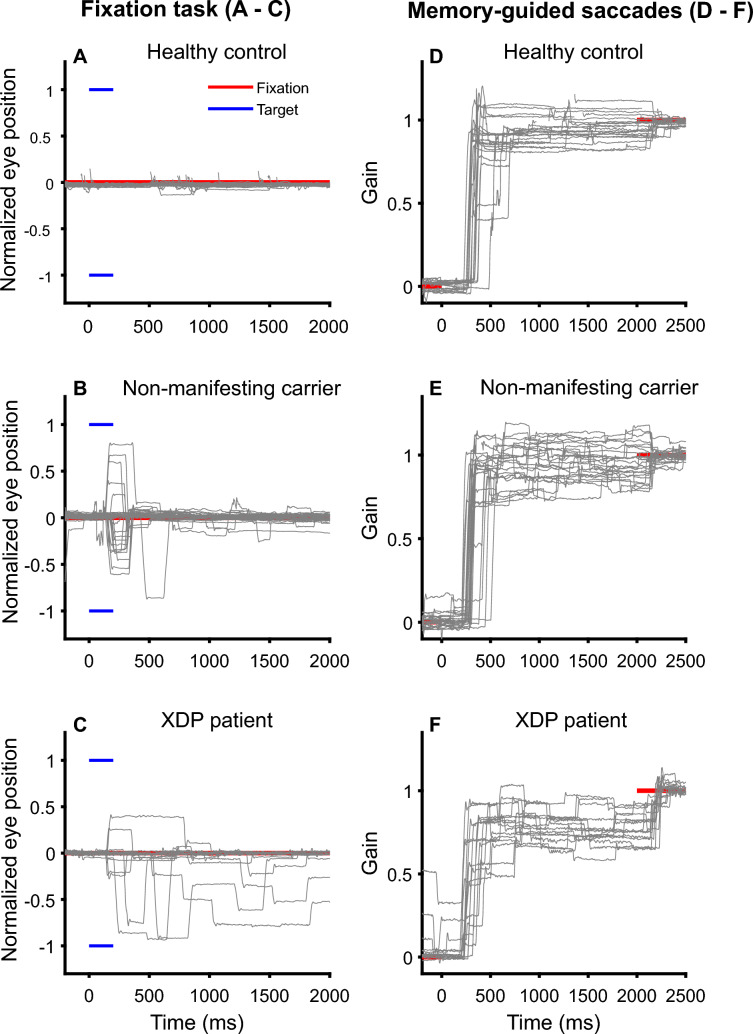
Representative horizontal eye position traces in MGS of a healthy control subject (top), non-manifesting carrier (middle row) and XDP patient (bottom) are shown for the fixation task (left side, **A–C**) during which subjects are instructed to look straight ahead (red line) but not to look towards the shortly presented peripheral visual target (blue). Normalized eye position (for 10° and 15° amplitude, **A–C**) and amplitudes of erroneously executed saccades are given relative to the target position (red line) as gain on the right side (**D–F**). The target was flashed for 200 ms on the left (− 1) or right (1) side. Note that the healthy subject maintained fixation by suppressing unwanted saccades to the visual targets. In contrast, XDP patients and NMC executed unwanted saccades towards the flashed target. Saccades towards the memorized target was typically performed with a multiple step pattern in XDP patients (saccade hypometria) which was only occasionally found in NMC. *MGS* memory-guided saccades, XD*P *X-linked dystonia-parkinsonism, *NMC* non-manifesting carriers

Based on the complexity of the volitional saccade tasks, we had to exclude three NMC, 11 XDP patients and four healthy control subjects due to insufficient comprehension of the anti-saccade, the memory-guided saccade (Fig. 2) or both tasks.

#### Memory-guided saccades

**Fig. 3:**
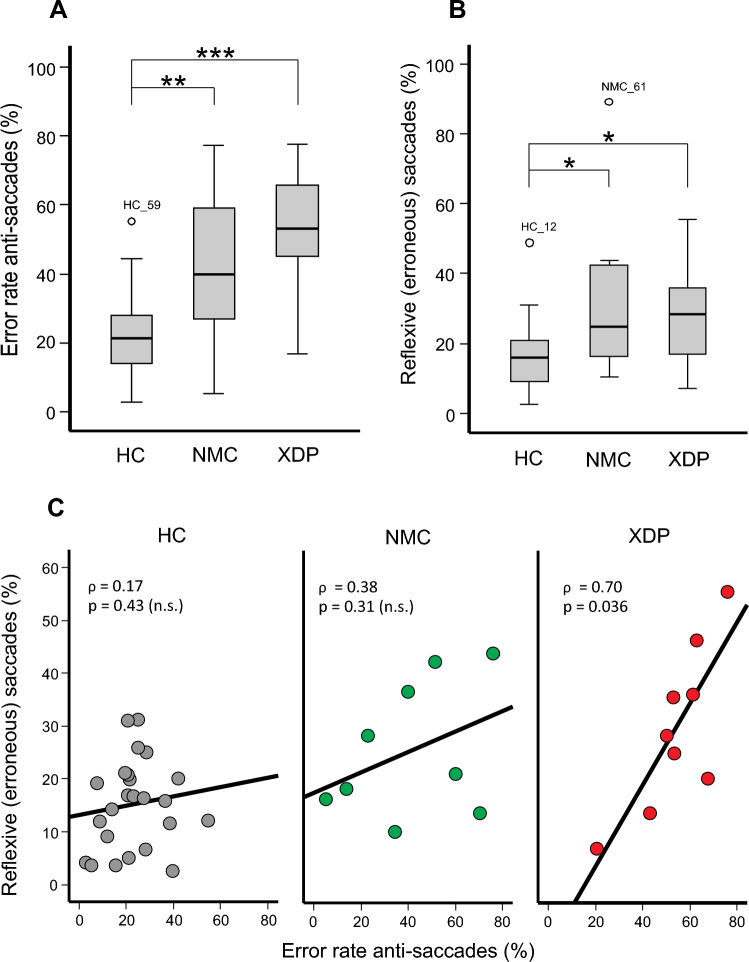
Error rates in anti-saccades (**A**) and MGS (**B**) in XDP patients, NMC, and HC. Not only XDP patients but also NMC showed higher error rates than HC. *p* values: * < 0.05; ** < 0.01, *** < 0.001. **C** Error rate (%) in the anti-saccade task significantly increase with the error rate in the memory-guided saccade task, i.e., erroneously performed reflexive saccades. There is a non-significant trend for this relation in NMC but not in HC. MGS memory-guided saccades, XD*P  *X-linked dystonia-parkinsonism, *NMC* non-manifesting carriers, *HC* healthy controls

The rate of erroneously performed reflexive saccades (RES) in the MGS task was different between groups (*Χ*^2^(2) = 7.806; *p = *0.020, Figs. 2, 3B). Not only XDP patients but also NMC had a larger RES (Fig. 2) compared to HC (XDP: *Z = *− 2.31; *p = *0.019; NMC: *Z = *− 2.16; *p = *0.031). XDP patients and NMC showed no difference (*p = *0.809). Individual performance regarding RES is shown in Supplementary Table 2.

The average latency of the first saccade for all memorization times (MT) was 420 ± 54 ms in XDP patients, 334 ± 54 ms in NMC and 328 ± 34 ms in HC. Although there was a significant main effect of MT (*F*(2,42) = 7.69, *p = *0.001) and an interaction of MT × group (*F*(4,68) = 2.96, *p = *0.032), there was no significant difference between groups in the MT categories after Bonferroni correction (*p > *0.112).

Rank-transformed data showed a main effect for MT (*F*(2,42) = 42.19, *p < *0.001) but no main effect for group and no interaction of group × MT. However, there was an interaction of MT and groups for saccade gain (*F*(3,38) = 2.751; *p = *0.043). It was smaller (hypometric) for XDP patients compared to NMC (*p < *0.001) and HC (*p < *0.001) (Fig. 1B). NMC and HC showed no difference (*p = *1.0). Saccade hypometria of XDP patients was found for each MT, compared to NMC (MT 2 s: *p < *0.02; MT 3 s, MT 4 s: *p < *0.001) and HC (MT 2 s – MT 4 s: *p < *0.001). NMC and HC did not differ in any MT (MT 2 s: *p = *0.343; MT 3 s: *p > *1.0; MT 4 s: *p = *0.582).

There was no interaction of gain and MT, i.e., there was no difference for XDP patients (*p > *1.0) nor for HC (*p* always > 0.41), irrespective of each single MT. NMC showed a difference between MT 2 s and MT 3 s (*p = *0.015) and MT 2 s and 4 s (*p = *0.03) but no difference between MT 3 s and MT 4 s (*p > *1.0).

Saccades towards the memorized target often (> 50%) showed a multi-step pattern in XDP patients and to a much lesser degree in NMC compared to HC (Fig. 2). The final eye position error (deviation of eye position from the previously presented target position) was small in all participants, irrespective of MT. There was a non-significant trend towards a larger variability of final eye position error in XDP. Since there was no interaction between groups and MTs regarding latency (*p = *0.519) and final eye position (*p = *0.367), the three MTs were averaged. The Kruskal–Wallis test showed no difference between groups in latency (*p = *0.676) or final eye position (*p = *0.344).

#### Anti-saccades

The error rate of anti-saccades (AER) showed a main effect between groups (*Χ*^2^(2) = 18.459; *p < *0.001, Fig. 3A). AER of XDP patients and NMC were larger compared to HC (XDP: *Z = *− 3.99; *p < *0.001; NMC: Z = − 2.70; *p = *0.021). There was no significant difference between XDP patients and NMC (*Z = *− 1.36; *p = *0.52). Supplementary Table 2 shows individual AER results of NMC. The average latency for correctly executed anti-saccades was longer in XDP patients (289 ms ± 11) compared to NMC and HC [(*F*(2,47) = 6.182, *p = *0.004); NMC: 249 ms ± 10, *p = *0.023, HC: 246 ms ± 7, *p = *0.004].

We tested for possible effects of fatigue during testing by comparing the first with the second half in each recording of pro- and anti-saccades (latency and gain). There was no main effect nor interaction in any of the comparisons (*p* always > 0.1).

#### Correlation of anti-saccades and memory-guided saccades

The AER of XDP patients correlated with the RES during MGS (*ρ* = 0.700; *p = *0.036, Fig. 3C). This did not apply for NMC (*p = *0.31) nor for HC (*p = *0.43). Note that cognitive test values (MOCA, MMSE, HADS, FAB) as well as clinical motor scores (BFMDRS / MDS-UPDRSIII) did not correlate with AER and RES in MGS (*p* always > 0.09, mean 0.50, SD = 0.23).

### Smooth pursuit eye movements

#### Sinusoidal smooth pursuit

**Fig. 4:**
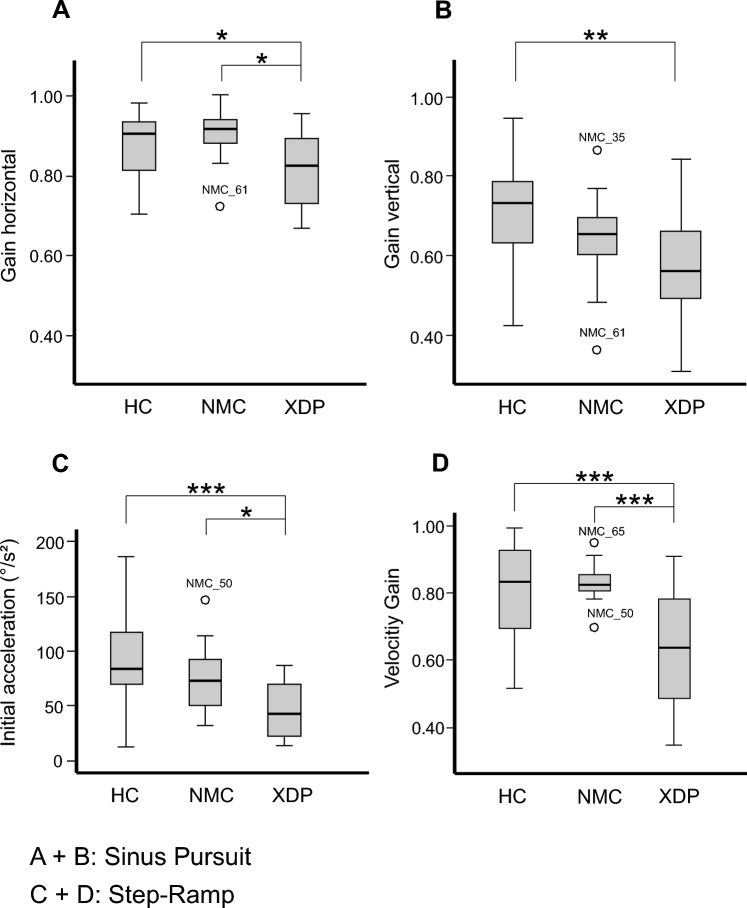
Sinusoidal SPEM in XDP patients, NMC, and HC. Horizontal (**A**) and vertical (**B**) velocity gain was decreased in XDP patients compared to NMC regarding horizontal gain and to HC regarding both. **C** and **D** Step-ramp pursuit movements. Initial acceleration was decreased in XDP patients compared to NMC and HC (**C**). **D** The velocity gain of foveopetal SPEM was significantly reduced in XDP patients, compared to NMC and HC. *p* values: * < 0.05; ** < 0.01, *** < 0.001. *SPEM*  smooth pursuit eye movements, XD*P *X-linked dystonia-parkinsonism, *NMC* non-manifesting carriers, *HC* healthy control subjects

We found a main effect for both horizontal (*Χ*^2^(2) = 6.7; *p = *0.035) and vertical (*Χ*^2^(2) = 8.895; *p = *0.012, Fig. 4A and B) smooth pursuit velocity gain between the groups. XDP patients had a significant smaller horizontal gain compared to NMC (*Z = *− 2.456; *p = *0.013) and HC (*Z = *− 2.049; *p = *0.04). Vertical pursuit gain was also smaller compared to HC (*Z = *− 2.803; *p = *0.005). The vertical SPEM gain was not significantly different between XDP patients and NMC (*p = *0.17). NMC and HC showed no difference considering horizontal (*p = *0.515) or vertical (*p = *0.114) gain. Correlation of clinical motor scores with horizontal and vertical gain revealed no significance for NMC (*p* always > 0.41), XDP patients (*p* always > 0.20) nor HC (*p* always > 0.25).

#### Foveopetal smooth eye pursuit

Latency of foveopetal SPEM did not show group differences (*p = *0.470). However, there was a main effect for the gain and the initial acceleration of foveopetal pursuit (gain: Χ^2^(2) = 10.903; *p = *0.004; inital acceleration: *Χ*^2^(2) = 12.445; *p = *0.002, Fig. 4C and D). XDP patients had lower pursuit gain compared to NMC (*Z = *− 3.106; *p = *0.001) and HC (*Z = *− 2.828; *p = *0.004). In addition, the initial acceleration was lower in XDP patients than in NMC (*Z = *− 2.523; *p = *0.011) and HC (*Z = *− 3.309; *p = *0.001). NMC and HC did not differ in gain (*p = *0.879) nor initial acceleration (*p = *0.260).

We detected no horizontal or vertical gaze-holding deficit, i.e., no gaze-evoked nystagmus and only very few square wave jerks (< 3% of NMC/XDP participants).

#### Relation of oculomotor and disease parameters

Neither latency and gain of pro-saccades, error rates of MGS or anti-saccades, nor SPEM velocity gain were correlated with the disease duration (symptom onset in XDP) or estimated age at disease onset or repeat number in NMC (*p* always > 0.29) and XDP subjects (*p* always > 0.09). Supplementary Table 2 displays NMC individual estimated age at disease onset and repeat number.

#### Correlation of abnormal oculomotor with imaging data

There was no correlation of the abnormal oculomotor findings in the NMC group with the abnormal striatal and pallidal brain regions described in our previous imaging study [19] that would have survived correction for multiple testing (*p* always > 0.28).

#### Correlation of abnormal oculomotor with gait parameters

Both oculomotor and gait abnormalities are subthreshold disorders which were identified by quantitative analysis only. Error rates of anti-saccades and memory-guided saccades were not correlated with the quantitative gait and stance parameters after correction for multiple testing (*p* always > 0.1).

## Discussion

In line with our main hypothesis, NMC already showed subtle oculomotor abnormalities compared to HC, i.e., before the onset of clinical symptoms or signs indicative of XDP (dystonia, parkinsonism). Interestingly, the error rates of anti-saccades and memory-guided saccades of NMC were already indistinguishable from symptomatic XDP patients reflecting pronounced fronto-striatal impairment as an early sign of XDP-related neurodegeneration.

This impairment of volitional saccades is also found in pre-manifest HD carriers [5] who—like our NMC [19]—show striatal atrophy prior to the manifesting phase of the disease [4, 26]. Saccade hypometria and smooth pursuit deficits were not found in NMC, in contrast to XDP patients of this study, an independent patient sample of our previous study [51], and in patients with PD [23, 25, 36]. In the following, we will compare oculomotor abnormalities in NMC with XDP, PD patients as well as pre-manifest and manifest HD.

### Pro-saccades

The latency of horizontal pro-saccades did not differ between XDP patients and NMC. This is different from HD, in which latency typically increases with a higher variability [39], even in the pre-manifest phase [5], highlighting a varying pattern of neurodegeneration. Latency of pro-saccades is mainly controlled by the PFC [43] and its projection areas, specifically to the superior colliculus (SC) in the dorsal midbrain. The SC itself is a crucial area in triggering volitional and visually guided saccades as it receives direct projections from the frontal and parietal cortex (e.g., frontal eye field, FEF; dorsolateral prefrontal cortex, DLPFC) and indirect projections via the basal ganglia circuit [31]. While the fronto-collicular pathways exert excitatory influence on the SC, the indirect pathways via the basal ganglia inhibit the SC, i.e., activity of SC relies on the net influence of these competing afferent projections. Latency is crucially context-dependent and depends on the type of saccade, specifically if they are reflexive or internally triggered [31].

To initiate a saccade, fixation-related activity in the SC must be suppressed [33]. The substantia nigra (pars reticulata, SNpr) and the globus pallidus internus (GPi) serve as inhibitory “gate keepers” of the SC. In striatal neurodegeneration in HD [39], the striatal control on the SNpr fails and subsequently reduces the normal inhibitory control of the SNpr on the SC leading to an impairment to initiate saccades (compensated by concomitant blinks or head thrusts), and to difficulties in suppressing saccades to novel visual stimuli and prolonged latency of saccades. Thus, increased saccade latencies could not only be related to prefrontal neurodegeneration and atrophy in HD but also additional striatal degeneration. In contrast to HD with excessively increased saccade latencies as well as considerable latency variability [39], pro-saccade latency of XDP patients did not differ from healthy control subjects but they showed an increased variability in latency. Pro-saccade latencies and variability of NMC were still within the range of HC. Although indistinguishable in horizontal saccade latency, both NMC and XDP patients had shorter latencies during small saccade amplitudes than HC which increased with larger amplitudes, similar to the amplitude-latency behavior in PD [12, 53, 57]. Thus, those fronto-collicular and fronto-striatal projections controlling saccade activity in SC to initiate saccades (latency) seem less impaired compared to HD patients and are rather in line with PD [49].

Saccade hypometria in XDP patients is probably due to caudate degeneration, favoring the indirect way of the basal ganglia on the SC [51]. Similar to PD [45], the deficient nigro-collicular inhibition leads to saccade hypometria in XDP in this and our previous patient cohort [51] but not in NMC of this study. The initial dystonic movements result from a striosomal loss projecting to the SNpr with a postulated increase of dopamine release [56], potentially activating the direct way of the basal ganglia. Possibly in NMC, caudate degeneration is already commencing but outweighed by the active direct way. Alternatively, saccade hypometria may result from cerebellar dysfunction as neurodegeneration has also been identified in the cerebellum in XDP patients [20]. Cerebellar degeneration, however, does not seem to be prominent in NMC as abnormal smooth pursuit eye movements and other cerebellar signs (gaze-holding function) were not found and imaging findings revealed no volumetric changes [19].

### Voluntary saccades (anti-saccades, memory-guided saccades)

The most striking finding in our NMC was the significant increases of the error rates in the anti-saccade and memory-guided saccade paradigms, similar to XDP patients. In fact, the increased error rates in the anti-saccade task of our NMC participants were indistinguishable from XDP patients of this and of our previous study [51]. This replication indicates increased AER to be a robust finding in XDP patients.

In the anti-saccade paradigm, the visual stimulus provokes a visually guided reflexive pro-saccade that needs to be suppressed. This is accomplished by a direct fronto-nigro-collicular pathway inhibiting the SC. Concomitantly, two other circuits involving the striatum with opposing excitatory and inhibitory pathways are activated which modify the net influence on the SNpr, thereby functionally disinhibiting the SC to facilitate volitional saccades [55]. The basal ganglia play thereby a crucial role in solving the opposing commands of volitional and reflexive (automatic) saccades. As a consequence, striatal neurodegeneration weakens the capacity of volitional saccadic signals (anti-saccades) to override automatic responses (visual reflexive pro-saccades). The striatal role, specifically of the GPi, in modifying the output of the SNpr on the SC becomes evident in PD patients with deep brain stimulation: the error rate significantly decreases with active GPi stimulation [2], in contrast to stimulation of the subthalamic nucleus.

This inhibitory gate function of the SNpr is highly challenged when the subject wants to perform a saccade in case immediate compelling information is not available. While the direct fronto (FEF)-collicular and parietal-collicular pathways determine the onset of saccades (when to start), the indirect pathways via the basal ganglia specify the context and nature of the target. Neural activity in the striatum depends on memory, expectation, and perceptual decisions [33].

The high AER in NMC and XDP patients could not only reflect striatal (and as an immediate consequence) but also prefrontal neurodegeneration (FEF, DLPFC). Lesions in the DLPFC lead to an increased error rate of anti-saccades as the DLPFC usually inhibits reflexive misdirected saccades [42]. Fixation neurons in the PFC may also be involved in the deficient inhibition of inappropriate/unwanted actions, i.e., the reflexive saccade in the anti-saccade paradigm [58].

The increased error rate in the memory-guided saccade (MGS) task could be related to striatal, parietal and prefrontal pathology. MGS are largely controlled by basal ganglia loops. Patients with lesions of the putamen generate regular saccades but exhibit deficits in saccade accuracy and error rates to remembered target locations, i.e., in MGS [54]. In PD, MGS are more strongly impaired than reflexive visually guided pro-saccades [53]. Striatal neurodegeneration in manifest [39] and pre-manifest HD [5] is also associated with grossly increased error rates of MGS. This excessive distractibility (directional error rates) in HD has been related to neurodegenerative involvement of the SNpr, releasing SC disinhibition [38].

Unlike PD and our XDP patients, we did not find an increased latency and hypometria of MGS in NMC. In addition, NMC showed fewer multi-step pattern of saccades towards a memorized stimulus as seen in PD, MSA-P and our XDP patients [57]. MSP is largely due to a dysfunction of the basal ganglia [22], favoring the indirect way, which is not yet prominently involved in NMC.

After triggering MGS by the FEF, the DLPFC and the posterior parietal cortex (PPC) with the parietal eye field (PEF) contribute to its saccade accuracy. The FEF is involved in the initiation of anti- or memory-guided saccades [43]. Short-term spatial memory in the MGS task is accomplished by the PPC and the DLPFC [41]. Once the target disappears in the MGS task, the DLPFC is activated and maintains target position until the target reappears [43]. As there was neither a difference in the saccade gain of MGS, irrespective of the duration of the memorization interval, nor in the final eye position error between NMC and HC, the DLPFC seems to be functionally intact in NMC. Saccade hypometria and prolonged latency in MGS in our XDP patients could point to additional extrastriatal impairment (e.g., DLPFC) but final eye position error did not differ from the HC.

Remarkably, the striatal and extrastriatal neurodegeneration (DLPFC, FEF, PEF) in NMC does not seem to be related until symptoms and signs of XDP can be clinically appreciated, as error rates of anti- and memory-guided saccades were only correlated in XDP patients with disease duration but not in NMC with time to the estimated age at onset. As NMC did not differ both in saccade latency and other global cognitive test parameters striatal rather than potential extrastriatal prefrontal neurodegeneration seems to account for the increased error rates in anti-saccades and MGS.

To summarize, the oculomotor behavior of reflexive and volitional saccades in NMC resemble XDP, PD, and HD patients by their increased error rate of anti-saccades and MGS (Table 2) [8, 25, 36, 37]. NMC participants differ from saccades in PD patients by their normal saccade gain of reflexive pro-saccades. They also differ from HD patients by their normal saccade latency and velocity. The saccadic behavior of NMC is also different from MSA-P patients as we did not find combined saccadic hypo- and hypermetria of reflexive saccades nor gaze-evoked nystagmus, or an increased number of square wave jerks [57].

**Table 2:** Oculomotor performance in PD, MSA-P, XDP, NMC and HD

Oculomotor task	Direction	Parameter	PD	MSA-P	XDP	NMC	HD
Pro-saccades	Horizontal	Latency	=	= /↑	= /↓	=	↑↑
		Gain	↓	↓	↓	=	=
		Velocity	=	=	=	=	↓
	Vertical	Latency	=	= /↑	= /↓	=	↑↑
		Gain	↓	↓	=	=	=
		Velocity	=	↓↓	=	=	↓↓
Anti-saccades	Horizontal	Error rate	= /↑	=	↑	↑	↑↑
Memory-guided saccades	Horizontal	Error rate	= /↑	=	↑	↑	↑↑
		Gain	↓	↓	↓	=	= /↓
Smooth pursuit	Horizontal	Gain	↓	↓	↓	=	↓
	Vertical	Gain	↓	↓	↓	=	↓

↓↓: highly reduced; ↓: reduced; = : equal; ↑: increased; ↑↑: highly increased; Adapted from [51]

### Smooth pursuit eye movements

NMC showed normal sinusoidal SPEM (indistinguishable from HC), in contrast to XDP patients who showed impaired SPEM with reduced velocity gain, in line with the XDP patients in our previous study [51]*.* Thus, abnormal SPEM seems to indicate a state rather than a trait sign of manifest XDP, comparable to *Parkin* mutation carriers [36]. Sinusoidal smooth pursuit, ensuring target visibility and providing a high degree of target predictability, is also impaired in PD patients with reduced velocity gain [23, 36] and additional interruption by anticipatory saccades [17]. As long as PD patients use ongoing visually guided motion, they can use prediction to maintain movement control as spatial memory is preserved in PD [15]. In the foveopetal task, where prediction is less available, initial acceleration of smooth pursuit was also normal in NMC and HC but significantly reduced in XDP patients.

SPEM are initiated in the FEF, driven by the motion-sensitive complex V5/MST being activated by retinal target motion related activity [16]. Higher cognitive control of SPEM, i.e., motor planning or prediction, is processed in the supplementary eye field and the DLPFC [35].

In contrast to XDP patients, these SPEM-related prefrontal regions do not seem to be impaired in the prodromal phase of XDP, i.e., our NMC. The basal ganglia, specifically the striatum [50], also provide an internal cue for the development of a preparatory activity for a given movement in the sequence of repetitive movements [34]. This anticipatory function was impaired in XDP patients but does not seem to be involved in the prodromal phase (NMC). PD patients are prone to exert deficits in pursuit preparation and execution involving extra-retinal drive mechanisms as target motion- related retinal pursuit is often still normal in early PD [14, 23]. Patients with striatal and cortical neurodegeneration in HD also show impaired target motion-related retinal smooth pursuit [30, 32] but extra-retinal predictive pursuit has not yet been examined.

MSA-P patients show an even more reduced smooth pursuit velocity [44, 57]. Accordingly, they show more often catch-up saccades towards target velocity while PD patients generate additional anticipatory saccades making it necessary to back up towards target velocity [17].

### Limitations

Nine XDP patients were treated with Levodopa but none of the NMC subjects. Accordingly, their increased error rates cannot be explained by medication. Statistically, we did not find an impact of L-Dopa medication on saccade performance in XDP subjects of this and our previous studies [51].

## Conclusion

The oculomotor phenotype of XDP patients [51] could be confirmed by our findings in this study, indicating a robust finding and highlighting the necessity for replication studies in independent cohorts. Remarkably, NMC showed impaired volitional (increased error rates) but largely preserved reflexive saccades and smooth pursuit. The impairment of volitional saccades is already progressed to a magnitude that is not dissociable from the XDP patients and similar to HD and advanced PD. Abnormal smooth pursuit indicates a state rather than trait sign of the mutation in XDP. In the absence of additional oculomotor signs of PD (pro-saccade hypometria, smooth pursuit deficit) or HD (saccade slowing, grossly increased latencies, smooth pursuit deficit), the increased error rates of anti-saccades and MGS appear to be an prodromal oculomotor phenotype of NMC and XDP reflecting prodromal fronto-striatal dysfunction.

We will monitor our NMC participants closely to see when they develop clinical signs of XDP. This will allow to estimate if the estimated age at disease onset was calculated correctly and how long eye movement abnormalities in XDP precede manifesting signs of XDP.

## Supplementary Information

Below is the link to the electronic supplementary material.

Supplementary file1 (DOCX 25 kb)

## Data Availability

The data this study is based on are available from the corresponding author, upon reasonable request.

## Abbreviations

AER: Anti-saccade error rate
ANOVA: Analysis of variance
BFMDRS: Burke–Fahn–Marsden–Dystonia Rating Scale
DLPFC: Dorsolateral prefrontal cortex
FAB: Frontal assessment battery
FEF: Frontal eye field
GPi: Globus pallidus internus
HADS_D/A: Hospital Anxiety and Depression Scale
HC: Healthy controls
HD: Huntington’s disease
MDS_UPDRS: Movement Disorder Society Revision of the Unified Parkinson’s Disease Rating Scale
MGS: Memory-guided saccade
MMSE: Mini-Mental State Examination
MOCA-P: Filipino version of the Montreal Cognitive Assessment
MSA-P: Multiple system atrophy with predominant parkinsonism
MST: Medial superior temporal area
MT: Memorization time
NMC: Non-manifesting TAF1 mutation carriers
PD: Parkinson's disease
PEF: Parietal eye field
PFC: Prefrontal cortex
PPC: Posterior parietal cortex
PSP: Progressive supranuclear palsy
RES: Rate of reflexive erroneous saccades
SC: Superior colliculus
SNpr: Substantia nigra pars reticulate
SPEM: Smooth pursuit eye movement
SVA: Short interspersed elements [SINE]–variable number of tandem repeats [VNTR]–Alu
TAF1: TATA-box-binding-associated-factor-1
XDP: X-Linked dystonia-parkinsonism

## References

[1] Antoniades CA, Demeyere N, Kennard C, Humphreys GW, Hu MT (2015). Antisaccades and executive dysfunction in early drug-naive Parkinson's disease: the discovery study. Mov Disord.

[2] Antoniades CA, Rebelo P, Kennard C, Aziz TZ, Green AL, FitzGerald JJ (2015). Pallidal deep brain stimulation improves higher control of the oculomotor system in Parkinson's disease. J Neurosci.

[3] Arasaratnam CJ, Singh-Bains MK, Waldvogel HJ, Faull RLM (2021). Neuroimaging and neuropathology studies of X-linked dystonia parkinsonism. Neurobiology of disease.

[4] Aylward EH, Sparks BF, Field KM, Yallapragada V, Shpritz BD, Rosenblatt A, Brandt J, Gourley LM, Liang K, Zhou H, Margolis RL, Ross CA (2004). Onset and rate of striatal atrophy in preclinical Huntington disease. Neurology.

[5] Blekher T, Johnson SA, Marshall J, White K, Hui S, Weaver M, Gray J, Yee R, Stout JC, Beristain X, Wojcieszek J, Foroud T (2006). Saccades in presymptomatic and early stages of Huntington disease. Neurology.

[6] Blood AJ, Waugh JL, Münte TF, Heldmann M, Domingo A, Klein C, Breiter HC, Lee LV, Rosales RL, Brüggemann N (2018). Increased insula-putamen connectivity in X-linked dystonia-parkinsonism. Neuroimage.

[7] Bragg DC, Mangkalaphiban K, Vaine CA, Kulkarni NJ, Shin D, Yadav R, Dhakal J, Ton M-L, Cheng A, Russo CT, Ang M, Acuña P, Go C, Franceour TN, Multhaupt-Buell T, Ito N, Müller U, Hendriks WT, Breakefield XO, Sharma N, Ozelius LJ (2017). Disease onset in X-linked dystonia-parkinsonism correlates with expansion of a hexameric repeat within an SVA retrotransposon in TAF1. Proc Natl Acad Sci.

[8] Briand KA, Strallow D, Hening W, Poizner H, Sereno AB (1999). Control of voluntary and reflexive saccades in Parkinson's disease. Exp Brain Res.

[9] Brüggemann N, Domingo A, Rasche D, Moll CKE, Rosales RL, Jamora RDG, Hanssen H, Münchau A, Graf J, Weissbach A, Tadic V, Diesta CC, Volkmann J, Kühn A, Münte TF, Tronnier V, Klein C (2019). Association of pallidal neurostimulation and outcome predictors with X-linked dystonia parkinsonism. JAMA Neurol.

[10] Brüggemann N, Heldmann M, Klein C, Domingo A, Rasche D, Tronnier V, Rosales RL, Jamora RDG, Lee LV, Münte TF (2016). Neuroanatomical changes extend beyond striatal atrophy in X-linked dystonia parkinsonism. Parkinsonism Relat Disord.

[11] Brüggemann N, Rosales RL, Waugh JL, Blood AJ, Domingo A, Heldmann M, Jamora RD, Münchau A, Münte TF, Lee LV, Buchmann I, Klein C (2017). Striatal dysfunction in X-linked dystonia-parkinsonism is associated with disease progression. Eur J Neurol.

[12] Chambers JM, Prescott TJ (2010). Response times for visually guided saccades in persons with Parkinson's disease: a meta-analytic review. Neuropsychologia.

[13] Dominguez JF, Ng AC, Poudel G, Stout JC, Churchyard A, Chua P, Egan GF, Georgiou-Karistianis N (2016). Iron accumulation in the basal ganglia in Huntington's disease: cross-sectional data from the IMAGE-HD study. J Neurol Neurosurg Psychiatry.

[14] Fukushima K, Fukushima J, Barnes GR (2017). Clinical application of eye movement tasks as an aid to understanding Parkinson's disease pathophysiology. Exp Brain Res.

[15] Fukushima K, Fukushima J, Warabi T, Barnes GR (2013). Cognitive processes involved in smooth pursuit eye movements: behavioral evidence, neural substrate and clinical correlation. Front Syst Neurosci.

[16] Fukushima K, Yamanobe T, Shinmei Y, Fukushima J, Kurkin S, Peterson BW (2002). Coding of smooth eye movements in three-dimensional space by frontal cortex. Nature.

[17] Gorges M, Pinkhardt EH, Kassubek J (2014). Alterations of eye movement control in neurodegenerative movement disorders. J Ophthalmol.

[18] Goto S, Lee LV, Munoz EL, Tooyama I, Tamiya G, Makino S, Ando S, Dantes MB, Yamada K, Matsumoto S, Shimazu H, Kuratsu J-I, Hirano A, Kaji R (2005). Functional anatomy of the basal ganglia in X-linked recessive dystonia-parkinsonism. Ann Neurol.

[19] Hanssen H, Diesta CC, Heldmann M, Dy J, Tantianpact J, Steinhardt J, Sauza R, Manalo HTS, Sprenger A, Reyes CJ, Tucazon R, Laabs BH, Domingo A, Rosales RL, Klein C, Münte TF, Westenberger A, Oropilla JQ, Brüggemann N (2023). Basal ganglia atrophy as a marker for prodromal X-linked Dystonia-parkinsonism. Ann Neurol.

[20] Hanssen H, Heldmann M, Prasuhn J, Tronnier V, Rasche D, Diesta CC, Domingo A, Rosales RL, Jamora RD, Klein C, Münte TF, Brüggemann N (2018). Basal ganglia and cerebellar pathology in X-linked dystonia-parkinsonism. Brain.

[21] Hanssen H, Prasuhn J, Heldmann M, Diesta CC, Domingo A, Göttlich M, Blood AJ, Rosales RL, Jamora RDG, Münte TF, Klein C, Brüggemann N (2019). Imaging gradual neurodegeneration in a basal ganglia model disease. Ann Neurol.

[22] Harting JK, Updyke BV (2006). Oculomotor-related pathways of the basal ganglia. Prog Brain Res.

[23] Helmchen C, Pohlmann J, Trillenberg P, Lencer R, Graf J, Sprenger A (2012). Role of anticipation and prediction in smooth pursuit eye movement control in Parkinson's disease. Mov Disord.

[24] Hernández IH, Cabrera JR, Santos-Galindo M, Sánchez-Martín M, Domínguez V, García-Escudero R, Pérez-Álvarez MJ, Pintado B, Lucas JJ (2020). Pathogenic SREK1 decrease in Huntington’s disease lowers TAF1 mimicking X-linked dystonia parkinsonism. Brain.

[25] Hertel S, Sprenger A, Klein C, Kömpf D, Helmchen C, Kimmig H (2009). Different saccadic abnormalities in PINK1 mutation carriers and in patients with non-genetic Parkinson's disease. J Neurol.

[26] Hobbs NZ, Barnes J, Frost C, Henley SMD, Wild EJ, Macdonald K, Barker RA, Scahill RI, Fox NC, Tabrizi SJ (2010). Onset and progression of pathologic atrophy in Huntington disease: a longitudinal MR imaging study. Am J Neuroradiol.

[27] Laabs B-H, Klein C, Pozojevic J, Domingo A, Brüggemann N, Grütz K, Rosales RL, Jamora RD, Saranza G, Diesta CCE, Wittig M, Schaake S, Dulovic-Mahlow M, Quismundo J, Otto P, Acuna P, Go C, Sharma N, Multhaupt-Buell T, Müller U, Hanssen H, Kilpert F, Franke A, Rolfs A, Bauer P, Dobričić V, Lohmann K, Ozelius LJ, Kaiser FJ, König IR, Westenberger A (2021). Identifying genetic modifiers of age-associated penetrance in X-linked dystonia-parkinsonism. Nat Commun.

[28] Lee LV, Maranon E, Demaisip C, Peralta O, Borres-Icasiano R, Arancillo J, Rivera C, Munoz E, Tan K, Reyes MT (2002). The natural history of sex-linked recessive dystonia parkinsonism of Panay, Philippines (XDP)*. Parkinsonism Relat Disord.

[29] Lee LV, Pascasio FM, Fuentes FD, Viterbo GH (1976). Torsion dystonia in Panay, Philippines. Adv Neurol.

[30] Lee SU, Kim JS, Yoo D, Kim A, Kim HJ, Choi JY, Park JY, Jeong SH, Kim JM, Park KW (2022). Ocular motor findings aid in differentiation of spinocerebellar ataxia type 17 from Huntington's disease. Cerebellum.

[31] Leigh RJ, Kennard C (2004). Using saccades as a research tool in the clinical neurosciences. Brain.

[32] Leigh RJ, Newman SA, Folstein SE, Lasker AG, Jensen BA (1983). Abnormal ocular motor control in Huntington's disease. Neurology.

[33] Leigh RJ, Zee DS (2015). The neurology of eye movements.

[34] Lekwuwa GU, Barnes GR (1996). Cerebral control of eye movements: I. The relationship between cerebral lesion sites and smooth pursuit deficits. Brain.

[35] Lencer R, Sprenger A, Trillenberg P, Klein C, Ettinger U (2019). Smooth eye movements in humans: smooth pursuit, optokinetic nystagmus and vestibular ocular reflex. Eye movement research: an introduction to its scientific foundations and applications.

[36] Machner B, Klein C, Sprenger A, Baumbach P, Pramstaller PP, Helmchen C, Heide W (2010). Eye movement disorders are different in Parkin-linked and idiopathic early-onset PD. Neurology.

[37] Nakamura T, Bronstein AM, Lueck C, Marsden CD, Rudge P (1994). Vestibular, cervical and visual remembered saccades in Parkinson's disease. Brain.

[38] Oyanagi K, Takeda S, Takahashi H, Ohama E, Ikuta F (1989). A quantitative investigation of the substantia Nigra in Huntington's disease. Ann Neurol.

[39] Peltsch A, Hoffman A, Armstrong I, Pari G, Munoz DP (2008). Saccadic impairments in Huntington's disease. Exp Brain Res.

[40] Petrozziello T, Mills AN, Vaine CA, Penney EB, Fernandez-Cerado C, Legarda GPA, Velasco-Andrada MS, Acuña PJ, Ang MA, Muñoz EL, Diesta CCE, Macalintal-Canlas R, Acuña-Sunshine G, Ozelius LJ, Sharma N, Bragg DC, Sadri-Vakili G (2020). Neuroinflammation and histone H3 citrullination are increased in X-linked dystonia parkinsonism post-mortem prefrontal cortex. Neurobiol Dis.

[41] Pierrot-Deseilligny C, Müri RM, Nyffeler T, Milea D (2005). The role of the human dorsolateral prefrontal cortex in ocular motor behavior. Ann N Y Acad Sci.

[42] Pierrot-Deseilligny C, Müri RM, Ploner CJ, Gaymard B, Demeret S, Rivaud-Pechoux S (2003). Decisional role of the dorsolateral prefrontal cortex in ocular motor behaviour. Brain.

[43] Pierrot-Deseilligny C, Ploner CJ, Müri RM, Gaymard B, Rivaud-Péchoux S (2002). Effects of cortical lesions on saccadic eye movements in humans. Ann N Y Acad Sci.

[44] Pinkhardt EH, Kassubek J, Süssmuth S, Ludolph AC, Becker W, Jürgens R (2009). Comparison of smooth pursuit eye movement deficits in multiple system atrophy and Parkinson's disease. J Neurol.

[45] Pretegiani E, Optican LM (2017). Eye movements in Parkinson's disease and inherited parkinsonian syndromes. Front Neurol.

[46] Rashbass C (1961). The relationship between saccadic and smooth tracking eye movements. J Physiol.

[47] Reetz K, Tadic V, Kasten M, Brüggemann N, Schmidt A, Hagenah J, Pramstaller PP, Ramirez A, Behrens MI, Siebner HR, Klein C, Binkofski F (2010). Structural imaging in the presymptomatic stage of genetically determined parkinsonism. Neurobiol Dis.

[48] Rosales RL (2010). X-linked dystonia parkinsonism: clinical phenotype, genetics and therapeutics. J Mov Disord.

[49] Rottach KG, Riley DE, DiScenna AO, Zivotofsky AZ, Leigh RJ (1996). Dynamic properties of horizontal and vertical eye movements in parkinsonian syndromes. Ann Neurol.

[50] Schultz W, Apicella P, Scarnati E, Ljungberg T (1992). Neuronal activity in monkey ventral striatum related to the expectation of reward. J Neurosci.

[51] Sprenger A, Hanssen H, Hagedorn I, Prasuhn J, Rosales RL, Jamora RDG, Diesta CC, Domingo A, Klein C, Brüggemann N, Helmchen C (2019). Eye movement deficits in X-linked dystonia-parkinsonism are related to striatal degeneration. Parkinsonism Relat Disord.

[52] Steinhardt J, Hanssen H, Heldmann M, Sprenger A, Laabs BH, Domingo A, Reyes CJ, Prasuhn J, Brand M, Rosales R, Münte TF, Klein C, Westenberger A, Oropilla JQ, Diesta C, Brüggemann N (2022). Prodromal X-linked dystonia-parkinsonism is characterized by a subclinical motor phenotype. Mov Disord.

[53] Terao Y, Fukuda H, Ugawa Y, Hikosaka O (2013). New perspectives on the pathophysiology of Parkinson's disease as assessed by saccade performance: a clinical review. Clin Neurophysiol.

[54] Vermersch AI, Müri RM, Rivaud S, Vidailhet M, Gaymard B, Agid Y, Pierrot-Deseilligny C (1996). Saccade disturbances after bilateral lentiform nucleus lesions in humans. J Neurol Neurosurg Psychiatry.

[55] Watanabe M, Munoz DP (2011). Probing basal ganglia functions by saccade eye movements. Eur J Neurosci.

[56] Weissbach A, Bäumer T, Rosales R, Lee LV, Brüggemann N, Domingo A, Westenberger A, Jamora RD, Diesta CC, Brandt V, Tadic V, Zittel S, Klein C, Münchau A (2015). Neurophysiological fingerprints of X-linked dystonia-parkinsonism: a model basal ganglia disease. Mov Disord.

[57] Zhou H, Wang X, Ma D, Jiang Y, Li F, Sun Y, Chen J, Sun W, Pinkhardt EH, Landwehrmeyer B, Ludolph A, Zhang L, Zhao G, Wang Z (2021). The differential diagnostic value of a battery of oculomotor evaluation in Parkinson's disease and multiple system atrophy. Brain Behav.

[58] Zhou X, Constantinidis C (2017). Fixation target representation in prefrontal cortex during the antisaccade task. J Neurophysiol.

