# Supplementary material for: Oculomotor abnormalities indicate early executive dysfunction in prodromal X-linked dystonia-parkinsonism (XDP)
Source: J Neurol. 2023 May 16;270(9):4262–75. doi: 10.1007/s00415-023-11761-8 (PMC10421788; doi:10.1007/s00415-023-11761-8)
Supplement: Supplementary file 1 — Supplementary file1 (DOCX 25 kb) [file 415_2023_11761_MOESM1_ESM.docx]

# Supplementary Data

## Material and Methods

### Demographic, genetic and clinical data

**Table 1: Demographic, genetic and clinical data of the study population**

| Parameter | HC | NMC | XDP | *p* (NMC vs. HC) | *p* (NMC vs. XDP) |
| --- | --- | --- | --- | --- | --- |
| N | 28 | 13 | 20 | --- | --- |
| Age | 34.4 (6.24) | 31.9 (5.21) | 38.8 (6.51) | 1.0 | **0.035** |
| eAAO | --- | 39.47 (9.07) | 34.61 (6.17) | --- | --- |
| RN | --- | 42.93 (5.22) | 45.38 (3.20) | --- | --- |
| BFMDRS | 0.33 (1.42) | 0.25 (1.42) | 27.00 (3.47) | 0.305 | **< 0.001** |
| MDS-UPDRS-III | 1.61 (1.51) | 1.88 (2.27) | 28.33 (3.70) | 0.318 | **< 0.001** |
| MoCa-P | 26.22 (0.78) | 25.88 (1.17) | 22.00 (1.92) | 0.158 | 0.531 |
| MMSE | 28.39 (0.38) | 28.50 (0.57) | 27.67 (0.93) | 0.668 | 0.664 |
| HADS-D | 4.33 (0.70) | 4.00 (1.05) | 7.33 (1.71) | 0.802 | 0.646 |
| HADS-A | 6.33 (0.72) | 4.50 (1.07) | 6.00 (1.75) | 0.163 | 0.753 |
| FAB | 17.24 (1.25) | 16.63 (0.92) | 15.50 (0.71) | 0.015 | 0.937 |

Group data is stated by mean and standard deviation (M (SD)). The last two columns state p values calculated using Mann-Whitney U test.

Abbreviations: BFMDRS = Burke–Fahn–Marsden–Dystonia Rating Scale, eAOO = estimated age at disease onset, FAB = Frontal Assessment Battery, HADS-D/A = Hospital Anxiety and Depression Scale, HC = healthy controls, MDS-UPDRS-III = Movement Disorder Society Revision of the Unified Parkinson’s Disease Rating Scale, MMSE = Mini Mental State Examination, MoCa-P = Philippino version of the Montreal Cognitive Assessment, NMC = non-manifesting TAF1 mutation carriers, RN = repeat number, XDP = X-linked dystonia-parkinsonism.

### Apparatus and Oculomotor tasks

Stimuli were presented using a 23.5“ monitor (model LS24F350FHEXXP, resolution 1920 x 1080 pixel, refresh rate 60 Hz; Samsung Inc., Suwon, South Korea) and eye movements were recorded at 500 Hz binocularly using a portable eyetracker (EyeLink Portable Duo version 6.12, SR Research Limited, Ontario, CA; corneal reflection and pupil tracking, ellipse method; spatial resolution 0.01° with standard filtering). Head tracking was performed using a sticker at the forehead. Participants placed their head on a chin-rest at a distance of 54 centimetres to the screen.

**The recording duration of each task was between one minute and 3.5 minutes. To prevent fatigue, the subjects were asked to close their eyes and relax between the recording sessions.**

Before recording, the system was calibrated using a built-in 13-step calibration and validation process. Additionally, we performed a 13-step calibration afterwards recording eye positions at defined positions **plus two sinusoidal smooth pursuit movements horizontally and vertically with an amplitude of +/-15° and a frequency of 0.2 Hz**.

**Next, the instruction for the anti-saccade task was performed by using pictograms and oral instruction in English. Most participants spoke English but some Tagalog or other Filipino languages only. In these cases native speakers from the staff of the Neurology Department of the Makati Medical Center served as translators. After oral instruction the participants practiced the anti-saccade task, at the beginning with finger-pointing support, later on the participants performed at least 20 practice trials on their own. In some cases the practice trials were repeated. Eleven subjects did not understand the task and were excluded from the analyses (see degrees of freedom in ANOVA results). The same instruction procedure was performed for the memory saccade task.**

Pre-processing of the data was performed by transformation of data from pixel to degrees of visual angle with no additional filtering. Eye velocity for sinusoidal smooth pursuit paradigms was computed using a 5-point central median difference algorithm and a 70 Hz Gaussian filter subsequently. For step-ramp paradigms a 9-point central median difference algorithm was used with a 30 Hz Gaussian filter.

The stimulus consisted of a red dot (size 0.5°) on a black background moving on the screen. A 5-40° amplitude was shown for horizontal saccades (36 trials) for main sequence computation. Reflexive horizontal and vertical pro-saccades consisted of 5, 10 and15° steps (30 trials each) and 10 and 15° steps for anti-saccades (40 trials), all with a gap of 200 ms at the onset of lateral target step.
Memory-guided saccades consisted of two blocks of 30 trials with horizontal saccades of 10 and 15° amplitude pseudo-randomized to left and right direction and memorization time of 2, 3 and 4 seconds. Target was flashed for 200 ms while the subject was asked to continue fixation of the straight ahead target during the variable memorization time. Execution time with no stimulus visible was 2 seconds and a presentation of the target right after for 1000 ms (**Fig.2**). Additional analysis was performed for voluntary saccades: latency for correct anti-saccades was calculated as well as the error rate for final eye position (eye end position / target end position) in memory-guided saccades (MGS). The error rate in both anti-saccades and MGS were correlated with each other and clinical assessment scores.

Smooth pursuit eye movements were examined by sinusoidal and step-ramp smooth pursuit tasks.

In the horizontal sinusoidal paradigm, the target moved six sweeps with an amplitude of ± 15° at 0.2 Hz frequency with a peak velocity of 18.8°/s. In the vertical condition target amplitude was 14.25° with 0.2 Hz frequency and 17.9°/s peak velocity.

In the step-ramp paradigm, foveofugal and foveopetal target movements were based on Rashbass, 1961. Twenty-eight randomized trials (8 foveofugal, 20 foveopetal) were presented with a horizontal target (15°/s velocity). In the foveofugal task, the participants fixed the central target for 1500 ms or 2000 ms. Next, the target leaped 2.4° to the left or right side and moved with a velocity of 15°/s for 1000 ms ramp-wise in the same direction. During the foveopetal task, after the 2.4° step, the target moved with a velocity of 15°/s for 1000 ms ramp-wise in the opposite direction. The target stayed 500 ms or 1000 ms on the spot. The foveofugal task was put in to ensure no habituation during the foveopetal task. In both tasks the participants were asked to fix and follow the target.

Results

*Voluntary saccades*

**Table 2:**

NMC's individual performance of voluntary saccades

| NMC | Age | eAAO | RN | RES (%) | AER (%) |
| --- | --- | --- | --- | --- | --- |
| 1 | 26.4 | 39.69 | 46 | 28.33 | 28.08 |
| 2 | 34.8 | 45.85 | 38 | 13.73 | 70.37 |
| 3 | 37.1 | 48.12 | 44 | 16.33 | 5.13 |
| 4 | 28.7 | 45.76 | 38 | 36.67 | 40.00 |
| 5 | 28.7 | 32.22 | 46 | 21.15 | 60.00 |
| 6 | 27.2 | 50.80 | 37 | 42.37 | 51.28 |
| 7 | 39.2 | 44.89 | 40 | 10.20 | 34.29 |
| 8 | 26.3 | 25.08 | 52 | 43.90 | 76.47 |
| 9 | 34.3 | - | 43 | 89.29 | 25.93 |
| 10 | 23.4 | 18.68 | 54 | 18.33 | 13.89 |

Abbreviations: AER = anti-saccade error rate, eAAO = estimated age at onset, NMC = non-manifesting TAF1 mutation carrier, RES = rate of reflexive erroneous saccades, RN = (hexametric) repeat number.
